# Methylglyoxal Induces Changes in the Glyoxalase System and Impairs Glutamate Uptake Activity in Primary Astrocytes

**DOI:** 10.1155/2017/9574201

**Published:** 2017-06-08

**Authors:** Fernanda Hansen, Fabiana Galland, Franciane Lirio, Daniela Fraga de Souza, Carollina Da Ré, Rafaela Ferreira Pacheco, Adriana Fernanda Vizuete, André Quincozes-Santos, Marina Concli Leite, Carlos-Alberto Gonçalves

**Affiliations:** ^1^Departamento de Bioquímica, Instituto de Ciências Básicas da Saúde, Universidade Federal do Rio Grande do Sul, 90035-003 Porto Alegre, RS, Brazil; ^2^Universidade Feevale, Rio Grande do Sul, 93525-075 Novo Hamburgo, RS, Brazil

## Abstract

The impairment of astrocyte functions is associated with diabetes mellitus and other neurodegenerative diseases. Astrocytes have been proposed to be essential cells for neuroprotection against elevated levels of methylglyoxal (MG), a highly reactive aldehyde derived from the glycolytic pathway. MG exposure impairs primary astrocyte viability, as evaluated by different assays, and these cells respond to MG elevation by increasing glyoxalase 1 activity and glutathione levels, which improve cell viability and survival. However, C6 glioma cells have shown strong signs of resistance against MG, without significant changes in the glyoxalase system. Results for aminoguanidine coincubation support the idea that MG toxicity is mediated by glycation. We found a significant decrease in glutamate uptake by astrocytes, without changes in the expression of the major transporters. Carbenoxolone, a nonspecific inhibitor of gap junctions, prevented the cytotoxicity induced by MG in astrocyte cultures. Thus, our data reinforce the idea that astrocyte viability depends on gap junctions and that the impairment induced by MG involves glutamate excitotoxicity. The astrocyte susceptibility to MG emphasizes the importance of this compound in neurodegenerative diseases, where the neuronal damage induced by MG may be aggravated by the commitment of the cells charged with MG clearance.

## 1. Introduction

Astrocytes are essential for the survival and activity of neurons in physiological and pathological conditions [1]. The key role of astrocytes in the brain includes defense against oxidative stress, regulation of the synthesis, release of glutathione (GSH), glutamate uptake, and the modulation of synaptic activity, amongst other mechanisms [2, 3]. Furthermore, the astrocyte network that is formed by gap junctions is essential to the ionic and metabolic homeostasis of the central nervous system, as astrocytes play a critical role in maintaining the homeostatic regulation of extracellular pH, K^+^, and glutamate levels [4].

Brain tissue is highly dependent on glucose, not only as an energetic source but also for the synthesis of glutamate and GSH. However, an elevated flow of glucose in the brain tissue, as occurs in diabetes mellitus, can lead to deviations from the glycolytic pathway, causing elevations in methylglyoxal (MG), an extremely reactive aldehyde that is involved in dicarbonyl stress and advanced glycation end product (AGE) formation from proteins, nucleic acids, and lipids [5]. In this context, the glyoxalase system is important for detoxifying MG and, therefore, for maintaining MG concentrations under control, in turn, preventing AGE formation [6].

We have investigated the effects of MG exposure on C6 glioma cells [7], acute brain slices [8], and on rat behavior in vivo [9]. However, only a few studies have investigated the effect of MG on isolated astrocytes and, consequently, on their specific activities. Data in the literature indicate that the impairment of astrocyte functions is linked to neurodegenerative diseases [10], such as Alzheimer's disease, where AGE accumulates in the neurons and astroglia, as well as in amyloid plaques [11]. The importance of astrocyte-mediated neuroprotection against elevated levels of MG has been proposed [3].

Although astrocytes have a more developed glyoxalase system than neurons, protecting them from dicarbonyl stress [12, 13], it has been suggested that astrocytes are also damaged by glycation processes in diabetes mellitus and neurodegenerative diseases [11, 14]. In fact, its direct effects on astrocytes could aggravate the neuronal damage that is induced by MG. We, herein, studied the direct susceptibility of primary astrocyte cultures from Wistar rats to MG exposure, evaluating specific changes in the glyoxalase system, GSH content, glutamate uptake, and gap junction activity. Moreover, we compared the results of primary astrocyte cultures with those of C6 glioma cells, assuming eventual differences between these two glial cultures.

## 2. Materials and Methods

### 2.1. Materials

Methylglyoxal (MG), propidium iodide, [3(4,5-dimethylthi-azol-2-yl)-2,5-diphenyl tetrazolium bromide] (MTT), neutral red, carbenoxolone disodium salt (CBX), aminoguanidine hemisulfate salt (AG), standard glutathione, o-phthaldialdehyde, metaphosphoric acid, L-glutamate, N-methyl-D-glucamine, 4-(2-hydroxyethyl) piperazine-L-ethanesulfonic acid (HEPES), and cell culture materials were purchased from Sigma (Saint Louis, MO, USA). Dulbecco's modified Eagle medium (DMEM) and Dulbecco's phosphate-buffered saline (DPBS) were purchased from Gibco BRL (Carlsbad, CA, USA). Fetal bovine serum was obtained from Cultilab (Campinas, SP, Brazil), and L-[2,3-^3^H] glutamate was purchased from Amersham International (United Kingdom). Polyclonal anti-EAAT1 (GLAST) and anti-EAAT2 (GLT-1) were purchased from Abcam (Cambridge, MA, USA), anti-EAAT3 was purchased from Novus Biologicals (Littleton, CO, USA), and polyclonal anti-glyoxalase 1 was purchased from Santa Cruz Biotechnology Inc. (Dallas, Texas, USA). All other chemicals were purchased from local commercial suppliers.

### 2.2. Primary Astrocyte Culture

Primary astrocyte cultures from Wistar rats were prepared as previously described [15]. Procedures were in accordance with the National Institutes of Health Guide for the Care and Use of Laboratory Animals and were approved by the local authorities. Briefly, the cerebral cortex of newborn Wistar rats (1-2 days old) were removed and mechanically dissociated in Ca^2+^- and Mg^2+^-free Dulbecco's phosphate-buffered saline (DPBS), pH 7.2, containing (in mM) 137.93 NaCl, 2.66 KCl, 8.09 Na_2_HPO_4_, 1.47 KH_2_PO_4_, and 5.55 glucose. The cortex was cleaned of meninges and mechanically dissociated by sequential passage through a Pasteur pipette. After centrifugation at 1400 rpm for 5 min, the pellet was resuspended in DMEM (pH 7.6) supplemented with 8.39 mM HEPES, 23.8 mM NaHCO_3_, 0.1% amphotericin B, 0.032% garamycin, and 10% fetal bovine serum. Approximately 800,000, 300,000, or 50,000 cells were seeded in each well in 12-, 24-, or 96-well plates, respectively, and maintained in DMEM containing 10% fetal bovine serum in 5% CO_2_/95% air at 37°C. Cell were then allowed to grow to confluence and used at 21 days. The medium was replaced by DMEM with 1% fetal bovine serum in the absence or presence of MG. CBX (0.1 mM) was added 15 min before MG exposure, and afterwards, cells were coincubated with MG at 24 h. Coincubation of MG and AG at 24 h was also performed.

### 2.3. C6 Glioma Cell Culture

The C6 glioma cell line, an astrocyte-like cell line, was obtained from the American Type Culture Collection (Rockville, Maryland, USA). Late passage cells, harvested after at least 100 passages, were seeded in 12-, 24-, or 96-well plates at densities of 20,000, 10,000, or 2000 cells/well, respectively, and cultured in DMEM (pH 7.6) supplemented with 5% fetal bovine serum, 2.5 mg/mL Fungizone, and 100 U/L gentamicin in 5% CO_2_/95% air at 37°C. After the cells had reached confluence, the culture medium was replaced by DMEM without serum in the absence or presence of MG [16]. CBX (0.1 mM) was added 15 min before MG exposure, and cells were subsequently coincubated with MG for 24 h. Coincubation of MG and AG for 24 h also was performed.

### 2.4. MTT Reduction Assay

Cells were treated with 0.5 mg/mL MTT for 30 min in 5% CO2/95% air at 37°C. The medium was then removed, and MTT crystals were dissolved in DMSO. Absorbance values were measured at 560 and 650 nm. The reduction of MTT was calculated by the following formula: [(abs 560 nm) − (abs 650 nm)] [17]. Results were expressed as percentages of the control.

### 2.5. Neutral Red Incorporation Assay

Cells were treated with 50 *μ*g/mL neutral red (NR) for 30 min in 5% CO_2_/95% air at 37°C. The cells were then rinsed twice with phosphate-buffered saline (PBS) for 5 min each time. The NR dye taken up by viable cells was then extracted with 500 *μ*L of acetic acid/ethanol/water (1/50/49, *v*/*v*). Absorbance values were measured at 560 nm [18]. Results were expressed as percentages of the control.

### 2.6. Propidium Iodide Uptake Assay

At the end of treatments, cells were incubated with 7.5 *μ*M propidium iodide (PI) for 15 min and viewed with a Nikon inverted microscope with a TE-FM Epi-Fluorescence accessory. Images were transferred to a computer with a digital camera [18]. Positive cells were quantified using ImageG® software.

### 2.7. Evaluation of Glyoxalase System

The astrocytes or C6 cultures were first lysed and homogenized in sodium phosphate buffer, pH 7.4. Cell homogenates were then centrifuged at 13000 rpm for 15 min at 4°C, and the supernatant was used for enzymatic activities and protein content measurements. Glyoxalase 1 activity was then determined as previously described [19] with some modifications. The assay was carried out in 96-well microplates using a microplate spectrophotometer (UV Star, Greiner). The reaction mixture (200 *μ*L/well) contained 50 mM sodium-phosphate buffer (pH 7.2), 2 mM MG, and 1 mM GSH (preincubated for 30 min at room temperature). Protein from the sample (10–20 *μ*g per well) was added to the buffer. The formation of S-(D)-lactoylglutathione was linear and monitored at 240 nm for 15 min at 25°C. A unit of glyoxalase I activity is defined as the amount of enzyme that catalyzes the formation of 1 *μ*mol of S-(D)-lactoylglutathione per minute. Specific activity was calculated and expressed as milliunits per milligram of protein or expressed as a percentage of the control.

### 2.8. Glutathione (GSH) Content Assay

Intracellular GSH levels (nmol/mg protein) were measured as previously described [20]. This assay detects only the reduced glutathione content. Cell homogenates were diluted in ten volumes of 100 mM sodium phosphate buffer, pH 8.0, containing 5 mM EDTA, and protein was precipitated with 1.7% metaphosphoric acid. Supernatant was assayed with o-phthaldialdehyde (1 mg/mL methanol) at room temperature for 15 min. Fluorescence was measured using excitation and emission wavelengths of 350 and 420 nm, respectively. A calibration curve was formed using standard GSH solutions (0–500 *μ*M). GSH concentration was expressed as the percentage of the control.

### 2.9. Glutamate Uptake Assay

Glutamate uptake was performed as previously described [21] with some modifications. Cells were briefly incubated at 37°C in a Hank's balanced salt solution (HBSS) containing (in mM) 137 NaCl, 5.36 KCl, 1.26 CaCl_2_, 0.41 MgSO_4_, 0.49 MgCl_2_, 0.63 Na_2_HPO_4_·7H_2_O, 0.44 KH_2_PO_4_, 4.17 NaHCO_3_, and 5.6 glucose, adjusted to pH 7.2. The assay was initiated by the addition of 0.1 mM L-glutamate and 0.33 *μ*Ci/mL L-[2,3-^3^H] glutamate. The incubation was stopped after 7 min for the astrocyte cultures and after 10 min for the C6 cultures by removing the medium and rinsing the cells twice with ice-cold HBSS. Cells were then lysed in a 0.5 M NaOH solution. Sodium-independent uptake was determined using N-methyl-D-glucamine instead of NaCl. Sodium-dependent glutamate uptake was obtained by subtracting the nonspecific uptake from the total uptake to obtain the specific uptake. Radioactivity was measured in a scintillation counter. Results were calculated as nmol/mg protein/min and were expressed as percentages of the control.

### 2.10. Protein Determination

Protein content was measured by Lowry's method using bovine serum albumin as standard [22].

### 2.11. Western Blot Analysis

After 24 h of MG addition, astrocytes or C6 cultures were processed for electrophoresis/Western blotting by directly homogenizing in electrophoresis sample buffer at pH 6.8 (containing 62.5 mM Tris-HCl, 2% (*w*/*v*) SDS, 5% (*w*/*v*) *β*-mercaptoethanol, 10% (*v*/*v*) glycerol, and 0.002% (*w*/*v*) bromophenol blue) and boiling for 5 min. Protein samples (15 *μ*g per lane), prepared for electrophoresis as described above, were analyzed by 12% sodium dodecyl sulfate polyacrylamide gel electrophoresis (SDS-PAGE) and transferred to nitrocellulose membranes using a semidry blotting apparatus (1.2 mA/cm^2^; 1 h) [23]. The membranes were blocked overnight or for 1 h at 4°C with 5% bovine BSA in Tris-buffered saline (TBS) (10 mM Tris, 150 mM NaCl, pH 7.5) and then incubated for 3 h with an anti-GLAST, anti-GLT1, or anti-EAAT3 antibody (diluted 1 : 1000, 1 : 200, 1 : 1000, resp., in TBS containing Tween-20 and 2% BSA), or overnight at 4°C with an anti-glyoxalase 1 antibody (diluted 1 : 10,000 in TBS containing Tween-20 and 2% BSA). Next, for anti-GLAST, anti-GLT1, or anti-EAAT3 antibody, membranes were incubated for 2 h at room temperature with horseradish peroxidase- (HRP-) conjugated anti-mouse secondary antibody. Next, for anti-glyoxalase 1, membranes were incubated for 1 h at 4°C with horseradish peroxidase- (HRP-) conjugated anti-rabbit secondary antibody (1 : 10,000). Equivalent loading for each sample was confirmed with antiactin (diluted 1 : 10,000 in TBS containing Tween-20 and 2% BSA). The chemiluminescent reactions were developed using luminol as the substrate (ECL Western Blotting Analysis System, GE Healthcare) and evaluated in the luminescence image analyzer (Image Quant LAS4000 from GE). The immunocontent of GLAST, GLT1, EAAT3, and glyoxalase 1 was determined for optical density. The bands were quantified using ImageJ software.

### 2.12. Statistical Analysis

Data are reported as the mean ± standard error and analyzed statistically by Student's *t*-test or by one-way ANOVA. Statistically significant one-way ANOVA was followed by a post hoc Dunnett's test. Data were considered significant when *p* < 0.05. All analyses were carried out using the SPSS software package or Prism 5.0 (GraphPad).

## 3. Results

### 3.1. Comparison of the Glyoxalase Systems in Primary Astrocytes and C6 Glioma Cells

**Figure 1:**
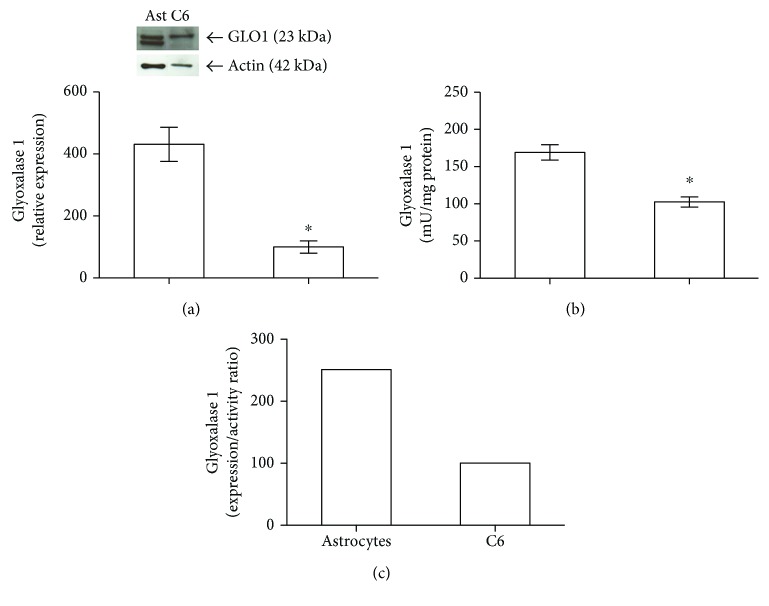
Glyoxalase 1 expression and activity are significantly higher in primary astrocytes than in C6 cells. Rat primary astrocyte cultures and C6 glioma cells were exposed to control conditions at 24 h. Glyoxalase 1 expression (a), glyoxalase 1 activity (b), and glyoxalase 1 expression/activity ratio (c). In (a) and (c), the control value of C6 cells is assumed as 100%. Each value represents mean ± standard error of three independent experiments performed in duplicate from Western blot analysis and six independent experiments performed in triplicate from glyoxalase 1 activity assays in both cell cultures. Insets are representative immunoblots for glyoxalase 1 (GLO1) or actin. GLO1 appears as a doublet band, in astrocyte samples, or a single band, in C6 samples, with an estimated size of approximately 23 kDa. Data were analyzed by Student's *t*-test, assuming *p* < 0.05 (a, b). ^∗^Significantly different from astrocytes.

Hypothesizing that astrocytes and C6 glioma cells may present metabolic differences, we measured the activity and content of glyoxalase 1, the rate-limiting enzyme of the glyoxalase system, in both cell types. The relative expressions and activities of glyoxalase 1 were significantly higher in astrocytes than in C6 cells (3.3- and 0.7-fold, resp.) (Figures 1(a) and 1(b)). Therefore, astrocytes present a 1.5-fold increase in glyoxalase 1 expression/activity ratio, when compared to C6 glioma cells (Figure 1(c)).

### 3.2. High Concentrations of MG Compromise the Cell Viability and Survival of Astrocytes and C6 Glioma Cells

**Figure 2:**
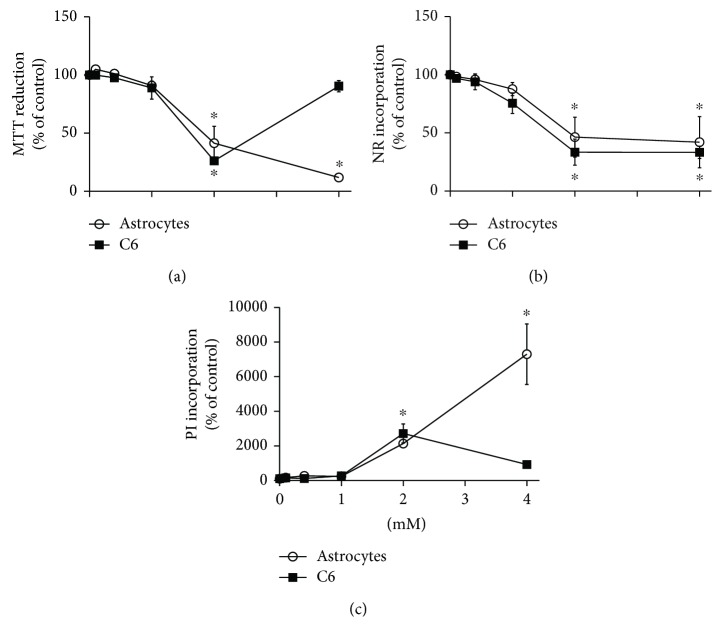
Effect of MG on cell viability and survival in astrocytes and C6 cells. MTT reduction (a), NR incorporation (b), and PI incorporation (c) were performed to assess cellular viability after 24 h of MG exposure. In the MTT reduction and NR incorporation assays, each value represents mean ± standard error of four independent experiments performed in triplicate from astrocytes and C6 cells, assuming the control value as 100%. Values of fluorescence intensity for PI/field are expressed as the mean ± standard error of three independent experiments performed in triplicate. Data from each cell culture were analyzed by one-way ANOVA followed by Dunnett's test, assuming *p* < 0.05. ^∗^Significantly different from control.

Astrocytes and C6 glioma cells were exposed to increasing concentrations of MG (from 0.1 to 4 mM) for 24 h; cell viability was then evaluated by MTT reduction, neutral red (NR) incorporation, and propidium iodide (PI) uptake assays (Figure 2). No impairment in MTT reduction capacity was observed up to 1 mM MG; however, 2 mM MG induced a significant decrease (about 50%) in cell viability in both cell types (Figure 2(a)). At the highest concentration of MG, we observed a greater decrease in MTT reduction in astrocytes, but curiously, we observed an increase in C6 glioma cells. In contrast, using the NR uptake assay, no impairment in cell viability was seen up to 1 mM, while the highest concentrations of MG significantly affected both cell types (Figure 2(b)). With regard to PI incorporation, neither of the cell types presented PI staining with up to 1 mM MG (Figure 2(c)); however, 2 mM MG induced a significant increase in uptake and, in astrocytes, the damage was higher at 4 mM MG. Interestingly, once again in C6 glioma cells, the highest concentration of MG cell survival apparently improved cell viability. Based on these assays, we chose intermediate concentrations of MG (0.4 or 1 mM) for further experiments.

### 3.3. High MG Increases Glyoxalase 1 Activity in Astrocytes

**Figure 3:**
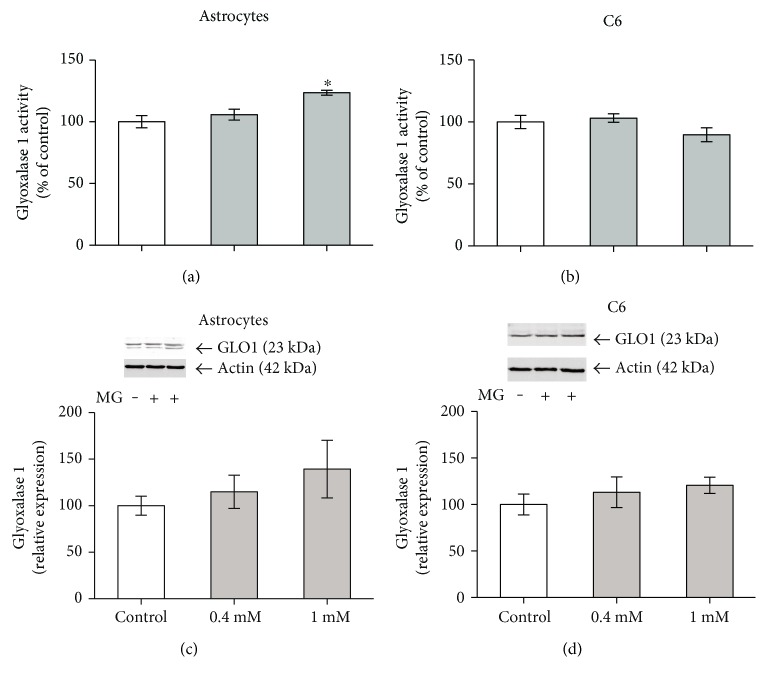
Effect of MG exposure on glyoxalase 1 activity and expression in astrocytes and C6 cultures. Rat primary astrocyte cultures and C6 glioma cells were exposed to 0.4 or 1 mM MG at 24 h. Glyoxalase 1 activity (a, b) and expression (c, d). Each value represents the mean ± standard error of five independent experiments performed in triplicate from the glyoxalase 1 activity assay and three independent experiments performed in duplicate from Western blot analysis in both cell cultures. Control value is assumed as 100%. Insets are representative immunoblots for glyoxalase 1 (GLO1) or actin. Data were analyzed by one-way ANOVA followed by Dunnett's test, assuming *p* < 0.05. ^∗^Significantly different from control.

The incubation of astrocyte cultures with 1 mM MG (but not 0.4 mM) for 24 h increased glyoxalase 1 activity (Figure 3(a)). This elevation was independent of the expression of glyoxalase 1 (Figure 3(c)). In contrast, we observed no changes in the activity or in the immunocontent of glyoxalase 1 in C6 glioma cells incubated with 0.4 or 1 mM MG (Figures 3(b) and 3(d)).

### 3.4. Elevated MG Augments Astrocyte GSH Content

**Figure 4:**
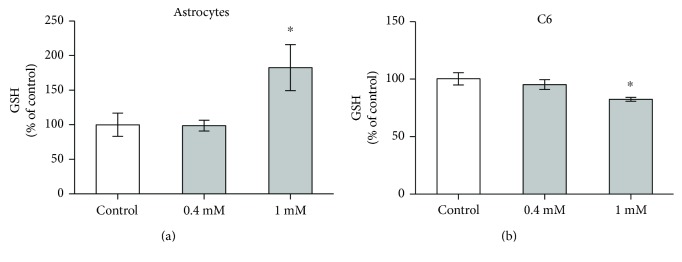
GSH content in astrocytes and C6 glioma cells after MG incubation. Rat primary astrocyte cultures and C6 glioma cells were exposed to 0.4 or 1 mM MG at 24 h. GSH content (a, b) is shown. Each value represents the mean ± standard error of six independent experiments performed in triplicate, assuming the control value as 100% for each type of cell culture. The mean absolute values of GSH in the control cell cultures were 38 and 39 nmol/mg protein in astrocytes and in C6 cells, respectively. Data were analyzed by one-way ANOVA followed by Dunnett's test, assuming *p* < 0.05. ^∗^Significantly different from control.

Considering the importance of GSH as a cosubstrate for glyoxalase 1 activity, we measured possible changes in GSH content induced by MG (Figure 4). Increased GSH content was induced by the incubation of astrocytes with 1 mM MG, but not with 0.4 mM MG (Figure 4(a)). Conversely, in C6 glioma cells, we observed a small but significant decrease in GSH content (Figure 4(b)).

### 3.5. Aminoguanidine Prevents the Effect of MG on Glyoxalase 1 Activity and GSH Content

In order to investigate whether the effects of 1 mM MG on glyoxalase 1 activity and GSH content is mediated by glycation, astrocytes were coincubated for 24 h with aminoguanidine (AG), a well-known antiglycation compound. AG per se did not alter glyoxalase 1 activity or GSH content (Table 1); however, this compound prevented the augmentations in glyoxalase 1 activity and GSH content.

### 3.6. MG Exposure Reduces Glutamate Uptake in Astrocytes, without Changing Glutamate Transporter Expression

**Figure 5:**
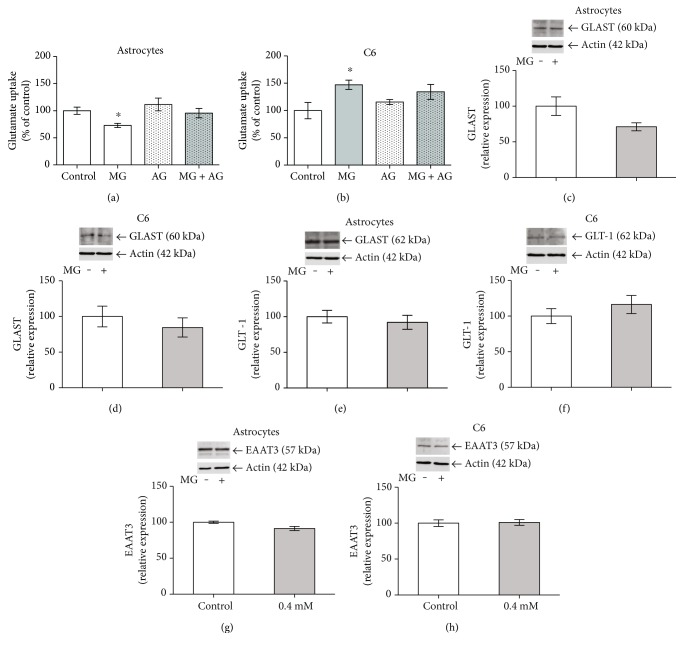
MG alters glutamate uptake in astrocytes and C6 cultures, but not GLAST, GLT-1, and EAAT3 expression. Rat primary astrocyte cultures and C6 glioma cells were exposed to 0.4 mM MG and 0.4 mM AG (a) at 24 h. Glutamate uptake (a, b), GLAST (c, d), GLT-1 (e, f), and EAAT3 (g, h) expressions are shown. In (a), each value represents mean ± standard error of ten independent experiments performed in triplicate with astrocytes, and in (b), six independent experiments performed in triplicate with C6 cells, assuming the control value as 100%. Data were analyzed by one-way ANOVA followed by Dunnett's test, assuming *p* < 0.05. ^∗^Significantly different from control. In (c)–(h), each value represents mean ± standard error of four independent experiments performed in duplicate, assuming the control value as 100%. Insets are representative immunoblots for GLAST, GLT-1, EAAT3, or actin. Data were analyzed by Student's *t*-test, assuming *p* < 0.05.

Astrocytes and C6 glioma cells were incubated with 0.4 mM MG (Figure 5) or 1 mM (data not shown) for 24 h. MG at both concentrations similarly decreased glutamate uptake activity (Figure 5(a)), and AG was able to prevent this decrease. Importantly, decrease glutamate uptake in astrocytes was not associated with changes in the contents of GLAST (Figure 5(c)), GLT-1 (Figure 5(e)), or EAAT3 (Figure 5(g)), as determined by Western blotting. Notice that an apparent decrease in the GLAST expression, observed in Figure 5(c), was not statistically significant (by Student's *t*-test, *p* = 0.11). On the other hand, in C6 glioma cells, MG at this concentration induced an increase in glutamate uptake activity (Figure 5(b)), but AG coincubation was also able to prevent this effect. No changes were observed in the content of glutamate transporters in C6 glioma cells: GLAST (Figure 5(d)), GLT-1 (Figure 5(f)), or EAAT3 (Figure 5(h)).

### 3.7. Carbenoxolone (CBX) Prevents MG Toxicity in Astrocytes

**Figure 6:**
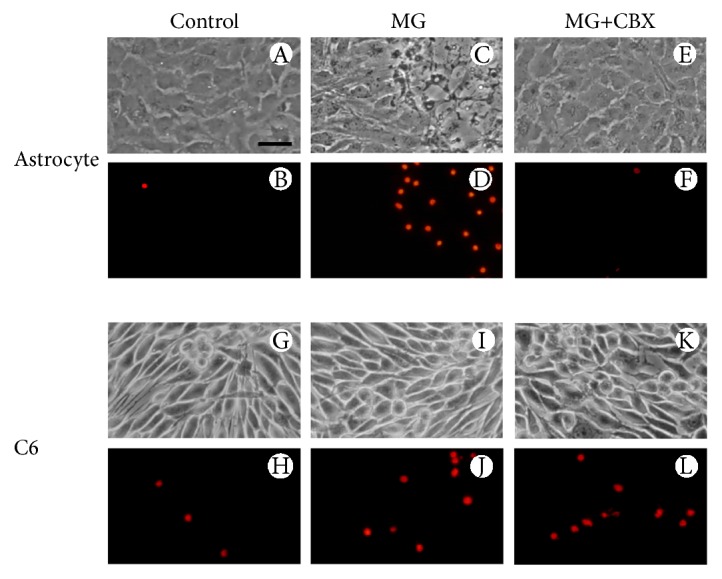
CBX protects astrocyte cultures from MG-induced cytotoxicity. Representative fields of astrocytes and C6 cultures treated with 1 mM MG for 24 h in the presence of PI. CBX was added 15 min before MG exposure, and cells were then coincubated with MG for 24 h. Phase contrast and nuclear fluorescent staining are shown under control conditions (A, B, G, H), MG exposure at 1 mM (C, D, I, J), and 1 mM MG + 0.1 mM CBX (E, F, K, L). All images are representative fields of at least three experiments, carried out in triplicate. Scale bar = 50 *μ*m.

Phase contrast and PI uptake fluorescent imaging were used to evaluate MG toxicity (at 1 mM) in primary astrocyte and C6 glioma cultures (Figure 6). PI-positive cell counting by microscopy showed increased staining of astrocytes (panels c and d) and C6 glioma cultures (panels i and j) following incubation with MG. Notice that we did not detect significant changes when mean PI uptake was measured by fluorimetry in the cell cultures incubated with MG at 1 mM, as shown in Figure 2(c). This apparent discrepancy reveals differences in the sensitivity and cell specificities with these different approaches in cell preparations.

As these cultures present significant differences in gap junction expression and function, we investigated whether the gap junctions could be involved in MG-induced alterations. In fact, CBX, a gap junction inhibitor was able to block the damage induced by MG (panel f), in astrocytes but not in C6 glioma cells (panel l).

## 4. Discussion

Our data showing the direct effect of MG on primary astrocytes indicate that these cells are very resistant to high concentrations of MG, when compared to neurons [12], possibly due to an efficient astrocyte glyoxalase system. Herein, we observed that glyoxalase 1 activity and expression are higher in astrocytes than in C6 glioma cells. The cytosolic glyoxalase system is responsible for preventing dicarbonyl stress and consists of two consecutive reactions; the first reaction is catalyzed by glyoxalase 1, in a rate-limiting step, where the substrate hemithioacetal, formed by nonenzymatic condensation of methylglyoxal and GSH, is converted into lactoylglutathione. In the second reaction, which is catalyzed by glyoxalase 2, GSH and lactate are released [6].

We found that the highest concentrations of MG significantly decreased the cell viability and survival of astrocyte cultures after 24 h of incubation. Therefore, for the 24 h assays, we chose high concentrations of MG concentrations (0.4 or 1 mM), but these were unable to compromise cell viability during this time. Moreover, similar nonphysiologically elevated concentrations of MG have been used in several other studies and models to investigate the glycation processes mediated by this compound [5, 12, 24–26]. Note that apparently MG does not affect cell viability linearly in C6 glioma cells. Based on MTT reduction and PI uptake assays, 4 mM MG is less toxic than 2 mM; however, the mechanism responsible for this unexpected result in C6 cells is unclear at the moment.

When we evaluated the glyoxalase 1 response to MG treatment, we observed an increase in activity in astrocytes at 1 mM that was not accompanied by any alteration in the enzyme's expression. MG, at this concentration, increased the GSH content and AG was able to block these increments, suggesting that astrocytes possess an important and responsive system to protect against MG toxicity. We are aware that large increases in MG levels (or low glyoxalase 2 activity) would result in S-(D)-lactoylglutathione accumulation, keeping GSH trapped and decreasing GSH availability [13]. However, in other studies of astrocyte cultures, 1-2 mM MG also increased intracellular GSH levels [12], and acrolein (a compound structurally related to MG) caused a biphasic response composed of an initial decrease in GSH content, followed by an increase 24 h afterwards [27] that was mediated by redox-sensitive signaling pathways (e.g., Nrf-2). Therefore, it is possible that MG, by activating such signaling pathways, could induce GSH increment at 24 h, as we observed. Accordingly, we did not find changes in reactive species formation, based on DCF levels at 24 h (data not shown).

In contrast, in C6 glioma cells, we observed a decrease in GSH levels upon MG exposure and no significant differences were observed in glyoxalase 1 activity and content. These findings, again, reinforce the important differences between primary astrocytes and C6 glioma cells, commonly used to investigate glial metabolism [16, 18]. In fact, astrocytes exhibit regional and functional heterogeneity in brain tissue, and cell culture studies can partially reflect this variability. We previously showed that incorporation of glycine in proteins was significantly decreased within 1 h and 3 h of MG exposure in C6 cells [7]. As glycine is involved in GSH synthesis [3], this explains, at least in part, the reduction in GSH in C6 cultures.

With regard to glutamate uptake, we found an impairment in glutamate uptake following MG treatment in astrocytes, but not in C6 cells, where an increase in glutamate uptake was observed. Accordingly, the exposure of astrocyte cultures to another glycating agent, glyoxal, caused a reduction in glutamate uptake activity and the formation of a GLT-1 CML adduct [26]. Data from our laboratory show that C6 cells cultured in high levels of glucose present increased glutamate uptake [28], while acute hippocampal slices exposed to MG present decreased glutamate uptake [8]. Together, these results suggest that alterations in glutamate uptake are an important target during MG glycation. However, at this moment, we do not know the mechanism(s) that mediate the changes in this activity. We herein evaluated transporters' expression only, but we are aware that these are modulated by posttranslational changes (e.g., [29]) that are sensitive to the redox environment (e.g., [30]), direct targets of glycation, and deserve further investigation. Results with AG suggest glycation induced by MG, but we cannot rule out alternative mechanisms.

Importantly, astrocytes and C6 glioma cell cultures exhibit differences in glutamate transporter expression. Astrocytes express and demonstrate functional activity, especially of GLAST and GLT-1, although they can also express EAAT3 [31]. On the other hand, although C6 cells also express GLAST and GLT-1 [32], they exhibit significant EAAT3 expression and activity, a characteristic of the neuronal glutamate transporter [28, 33]. Based on Western blotting analysis for these glutamate transporters (see Figure 5), we found higher levels of GLAST (75%), GLT-1 (50%), and even EAAT3 (26%) in astrocytes than in C6 cells. However, this approach does not allow us to evaluate the relative contribution of each EAAT to the general cell glutamate uptake. It is possible that the different structural specificities of these transporters and the different susceptibilities of astrocytes and C6 cells to MG-induced glycation may explain, in part, the differences between these cells. However, at this time, no experimental data support this idea.

Coincubation with AG prevented the MG-induced change in glutamate uptake activity in astrocytes and C6 glioma cells cultures, indicating that these alterations are possibly mediated by glycation. These functional alterations in glutamate transporters are not related to glutamate transporter expression, as MG exposure did not modify the protein expressions of GLAST, GLT-1, and EAAT3 by cells. It is possible that MG-induced glycation does not alter protein turnover within 24 h. Moreover, astrocyte glutamate transporters were not affected in an animal model of diabetes [34], where high levels of MG may be found due to hyperglycemia.

The ability of MG to decrease glutamate uptake in astrocyte-containing preparations is clear; however, the mechanism by which this occurs requires further comprehension. Although, direct glycation appears to be involved in astrocyte cultures, in hippocampal slices, the decrease in glutamate uptake was not prevented by AG incubation [8]. The current consensus is that astrocytes sense, integrate, and respond to stimuli generated by neurons or neural injury and that this response involves gap junctions. This idea is supported by the fact that gap junction inhibitors increase neuronal vulnerability when cocultures of astrocytes and neurons are exposed to oxidative stress [35] or to high glutamate [36]. On the other hand, considering the astrocytic syncytium in the brain, gap junction uncoupling could limit the extension of a lesion [37]. In the present study, we investigated whether CBX, a general blocker of gap junctions, would affect the damage induced by MG. CBX prevented the cytotoxicity induced by MG in astrocyte cultures. Expectedly, no changes in PI assay were observed when C6 glioma cells were exposed to MG and coincubated with the gap junction inhibitor [18]. These data indicate that gap junctions have a pivotal role in MG-induced damage in astrocytes and reinforce the idea that the impairment of astrocyte viability, induced by MG, involves glutamate excitotoxicity [36], supported by reduced glutamate uptake.

On the other hand, studies in animal models of diabetes and in vitro experiments that have determined the effects of high glucose levels on astrocytes' cultures [38, 39], as well as the AGE-albumin treatment of aortic endothelial cells [40], demonstrate that these compounds reduce gap junction communications. Although it is clear that the glycation process is involved in the mediation of MG astrocyte toxicity in cultures, it is unclear, at this moment, whether this involves AGE/RAGE signaling. This issue deserves further investigation in models of long-term MG exposure.

It is important to mention some limitations in this study. Firstly, we used a single commercial source of MG, and possible impurities may compromise findings. Secondly, we are aware that supraphysiological levels of MG limit the relevance of these results. In this context, further experiments using glyoxalase 1 silencing, as performed in other studies [12], would complement our understanding of the glyoxalase system. Finally, we focused on the effect of MG on glyoxalase 1 activity and quantity, but the effect on glyoxalase 2 also requires investigation, particularly on D-lactate levels, in order to obtain a clearer picture. Regardless of these limitations, our results contribute to our understanding regarding MG biochemistry and toxicity.

**Figure 7:**
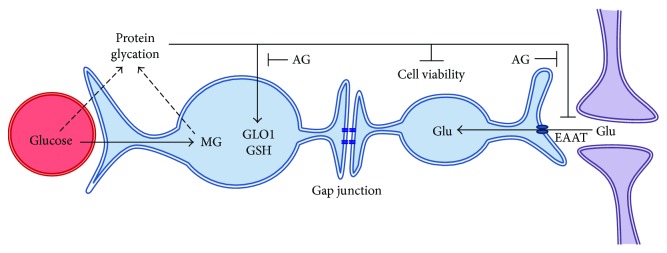
Schematic representation of the effect of methylglyoxal (MG) on astrocytes. Methylglyoxal (MG), a glycolytic derivative, causes protein glycation. Glyoxalase 1 (GLO1) activity and glutathione (GSH) levels increased following MG exposure, and glutamate (Glu) uptake activity is decreased. These alterations are blocked by aminoguanidine (AG). A decrease in cell viability is also observed, which is mediated by gap junctions.

A schematic representation of our results in primary astrocytes is shown in Figure 7, where MG (a glycolytic derivative) causes protein glycation, in turn, leading to an astrocyte response (increase in glyoxalase 1 activity and glutathione levels). On the other hand, signals of astrocyte suffering are indicated by decreased cell viability (as demonstrated by MTT reduction assay, NR uptake, and PI incorporation) and altered glutamate uptake activity, which is also mediated by gap junctions, as represented in Figure 7.

## 5. Conclusions

MG exposure impairs primary astrocyte viability, as evaluated by different assays. Astrocytes respond acutely to MG elevation by increasing the activity of glyoxalase 1 and GSH levels. This response, in part, may sustain cell viability and survival. In contrast, C6 glioma cells show signs of resistance against MG without significant changes in the glyoxalase system. AG coincubation provided evidence that MG toxicity is mediated by glycation. Glutamate uptake activity is an important target of MG glycation, and a significant decrease in glutamate transport is observed in astrocytes, without associated changes in the protein expression of major glutamate transporters. CBX, a general gap junction inhibitor, prevented the cytotoxicity induced by MG in astrocyte cultures. Therefore, our data reinforce the idea that astrocyte viability depends on gap junctions and that impairments induced by MG involve glutamate excitotoxicity. The susceptibility of astrocytes to MG emphasizes the importance of this compound in the brain reactions to neurodegenerative diseases, where neuronal damage induced by MG may be aggravated by the commitment of the cells responsible for MG clearance.

## Figures and Tables

**Table 1:** AG blocks the increments in glyoxalase 1 activity and GSH content following 1 mM MG.

	Astrocytes	
Treatment	Glyoxalase 1 activity	GSH content
Control	100.0 ± 5.0	100.0 ± 16.7
MG	123.6 ± 2.0^∗^	182.7 ± 33.3^∗^
AG	109.2 ± 3.4	80.9 ± 15.5
MG + AG	106.9 ± 3.4	93.6 ± 3.5

Rat primary astrocyte cultures were exposed to 1 mM MG and 0.4 mM AG at 24 h. Percentages of glyoxalase 1 activity and GSH content are shown. Each value represents mean ± standard error of five independent experiments performed in triplicate, assuming the control value as 100%. Data were analyzed by one-way ANOVA followed by Dunnett's test, assuming *p* < 0.05. ^∗^Significantly different from control.
